# Psychological reactions to COVID-19: Survey data assessing perceived susceptibility, distress, mindfulness, and preventive health behaviors

**DOI:** 10.1016/j.dib.2020.106687

**Published:** 2020-12-30

**Authors:** William H. O'Brien, Shan Wang, Huanzhen Xu, Shiwei Wang, Zaiying Yang, Joy Ting Yang, Qinwanxian Liu, Xin Zhang, Lingli Tang, Aniko V. Varga, Tracy Sims, Chung Xiann Lim, Somboon Jarukasemthawee, Kullaya Pisitsungkagarn

**Affiliations:** a Department of Psychology, Bowling Green State University, Bowling Green, OH 43403, United States; b Department of Behavioral Science, Duke Kunshan University, Kunshan, Jiangsu Province, China; c Department of Psychology and Cognitive Science, East China Normal University, Putuo, Shanghai, China; d Department of Psychology, Chulalongkorn University, Pathum Wan District, Bangkok, Thailand

**Keywords:** Perceived susceptibility to COVID-19, COVID-19 preventive health behavior, Post traumatic stress and physical symptoms related to COVID-19, Mindfulness and acceptance

## Abstract

The COVID-19 pandemic created a complex psychological environment for persons in America. A total of 450 USA MTurk workers completed measures of: (a) basic demographic characteristics; (b) health risk factors for COVID-19; (c) perceived susceptibility variables related to COVID-19; (d) COVID-19 preventive health behaviors; and (e) distress, physical symptoms, and quality of life measures. The surveys were completed between April 9, 2020 and April 18, 2020. This recruitment period corresponded to the first 2–3 weeks of lockdown in most of the USA. Follow-up surveys were completed by 151 of the USA participants between June 19, 2020 and July 11, 2020 (approximately 2 months after the first measurement).

These data permit evaluation of relationships among demographic variables, COVID-19 stress and coping, COVID-19 preventive health behavior, and the role of mindfulness as a possible moderator of distress as well as a predictor of preventive health behavior. The availability of follow-up data permit longitudinal analyses that provide a stronger basis for causal inference.

**Specifications Table**

**Table:** 

Subject	Psychiatry and Mental Health
Specific subject area	Measurement perceived objective risk, perceived susceptibility, physical symptoms, post traumatic symptoms, preventive health behavior related to COVID-19.
Type of data	Tables. Demographic data and measure summary data (measure subscale and total scores).
How data were acquired	Data were acquired using a multi-item survey that was posted online on MTurk. Two versions (Time 1 and Time 2) of the survey were used. Both surveys are uploaded to *Data in Brief* as supplementary files. The first author can also be contacted for a copy of the surveys.
Data format	Raw Data is available from the first author as an SPSS.SAV or Excel file. Data referred to in this paper are available to the public on Harvard Dataverse (Time 1 dataset: https://doi.org/10.7910/DVN/UDHBOB), Time 1 – Time 2 dataset: https://doi.org/10.7910/DVN/LIDGNS)
Parameters for data collection	Data were collected online using Qualtrics. All data were collected anonymously.
Description of data collection	On April 9, 2020, an announcement describing the study was posted on Amazon Mechanical Turk. Interested participants clicked on a link that provided a consent form. If participants electronically signed the consent form, they were then linked to the survey. The survey allowed them to complete items anonymously on Qualtrics. Participants received $1.00 for completing the survey. The survey link was kept active until April 18, 2020. After data cleaning 450 participants were kept in the dataset. These participant survey responses are located in the “Time 1 Dataset.” On June 19, 2020, all of the participants who responded to the Time 1 survey were sent an announcement asking them to complete a follow-up Qualtrics survey. After providing consent, they were directed to the follow-up survey. After data cleaning and matching with Time 1 responses, 151 participants were kept in this dataset. These participant survey responses are located in the “Time 1-Time 2 Dataset.” The survey can be obtained from the primary author. Figures 1 and 2 depict the geographical location of participants in the Time 1 Dataset and the Time 1-Time 2 Dataset. Tables 1 through 4 provide psychometric and descriptive statistics for the items and measures contained in the Time 1 and Time 1 – Time 2 datasets.
Data source location	Data source location: William H. O'Brien, Ph.D. Department of Psychology, Bowling Green State University, Bowling Green, Ohio 43402, USA.
Data accessibility	Repository name: Harvard Dataverse
	Time 1 Data identification number:
	UNF:6:NByE2zYH4xRsgUbD8vqE0A==
	Time 1 Direct URL to data:
	https://doi.org/10.7910/DVN/UDHBOB
	Time 1 – Time 2: identification number: UNF:6:MDiBgDZPE1mJMITo5PAb9A== Time 1 – Time 2 Direct URL to data:

## Value of the Data

• Data provide information on demographic characteristics, risk for COVID-19, perceived risk, distress, moderators of distress, and preventive health behaviors that allow for theoretically-based predictions of COVID-19 relevant outcomes.
• Data can be used for structural equation modeling and complex model testing of preventive health behaviors and psychological coping with the COVID-19 pandemic.
• Follow-up data were collected two months after the first measurement in the USA which permits measurement of change across time.

## 1 Data Description

The data are available as SPSS.SAV files. Both sets of data contain the constructs and measures variables listed in Table 1. The demographic characteristics of the two datasets are presented in Table 2. The descriptive statistics of the survey measures the two datasets are presented in Table 3.

**Table 1:** Constructs and measure description for Time 1 Dataset and Time 1 – Time 2 Dataset.

Construct	Measure	Sample Item	Number of Items	Cronbach's Alpha at Time 1	Cronbach's Alpha Time 1 – Time 2 Dataset
Mindfulness 1. Describe 2. Awareness 3. Observe 4. Nonreactivity 5. Nonjudgment	Five Factor Mindfulness Questionnaire	1. “I'm good at finding the words to describe my feelings,” 2. “I notice the smells and aromas of things,” 3. “I watch my feelings without getting carried away by them,” 4. “Usually when I have distressing thoughts or images I can just notice them without reacting,” 5. “I tell myself I shouldn't be thinking the way I'm thinking.”	24	.79	.89
Psychological Symptoms of Distress	General Health Questionnaire – Negative Items	“How often have you Felt constantly under strain?”	6		.90
Post-Trauma Stress	Impact of Events Scale	“I had waves of strong feelings about it.”	22	.97	.98
Intolerance of Uncertainty	Intolerance of Uncertainty Inventory	“Unforeseen events upset me greatly.”	12	.91	.94
Physical Symptoms	Patient Health Questionnaire	“Over the past week how often have you been bothered by headaches?”	15	.92	.80
Perceived Susceptibility to COVID-19	Single item created for study	“How likely is it that you will contract COVID-19?”	1	NA	NA
General Perceived Vulnerability to Disease	Perceived Vulnerability to Disease	“I have a history of susceptibility to infectious diseases.”	15	.82	.83
COVID-19 Preventive Health Behaviors – Personal Protective Equipment Use.	Preventive Action Taken Scale	“I wear a face mask outside of my home.”	3	.71	.73
COVID-19 Preventive Health Behaviors – Avoid Travel and Contact with Other People	Preventive Action Taken Scale	“I avoid public events and crowded places.”	5	.76	.84
General Quality of Life	Quality of Life Inventory	“How would you rate your quality of life?”	10	.83	.79

**Table 2:** Demographic characteristics of Time 1 Dataset and the Time 1 – Time 2 Dataset.

	Time 1 Dataset (*n* = 450)			Time 1 – Time 2 Dataset (*n* = 151)		
Variable	M	SD	%	M	SD	%
**Age**	36.68	11.27		37.98	11.93	
**Gender** Female Male			38 62			42 58
**Marital Status** Single Married Cohabitating In Long Term Relationship, Not Cohabitating Divorced Widowed			20 69 5 3 1 1			23 62 7 3 5 0
**Employment Status Before COVID-19** Employed 1–23 h/week Employed 24–39 h/week Employed ≥ 40 h/week Not employed/looking Not employed/not looking Retired Disabled			16 24 53 2 2 1 1			8 22 62 2 5 1 1
**Employment Status After COVID-19** Employed 1–23 h/week Employed 24–39 h/week Employed > 40 h/week Not employed/looking Not employed/not looking Retired Disabled			22 30 36 5 5 1 2			15 22 47 5 9 1 1
**Race/Ethnicity** Hispanic/Latinx White Black/African American Asian Pacific Islander American Indian or Alaska Native Two or more Unidentified			11 55 23 4 1 1 3 2			4 60 25 7 0 1 3 1
**Educational Attainment** High School Some College Associates Degree Bachelors Degree Masters Degree Beyond Masters			4 8 6 56 35 2			8 13 10 42 23 5
**Religious Affiliation** Catholic Protestant Jewish Muslim Buddhist Taoist Hindu Agnostic Atheist Nothing in Particular Other			18 53 3 2 1 1 1 6 5 6 4			23 34 4 2 2 1 1 9 9 10 6
Number of Children	1.19	1.00		1.09	1.19	
Annual Income in Dollars	66,226	68,515		NA^a^	NA^a^	
BMI	NA^b^	NA^b^		25.65	6.07	
NonCOVID-19 Illness (yes)			15			17
Taking Medication (yes)			15			18
In Isolation (Yes)			69			53
Number of Days Leave Home	2.42	.3.47		1.51	1.60	
Took COVID-19 PCR Test			NA^b^			8
Took COVID-19 Antibody Test			NA^b^			8
Currently COVID-19 Infected (confirmed by test)			NA^b^			5
COVID-19 infected in the past (confirmed by Test)			NA^b^			1

Superscript

a indicates item changed from Wave 1 to follow-up and superscript.

b indicates measure not collected at baseline.

**Table 3:** Descriptive statistics of the Time 1 Dataset (n = 450).

Measure	*M*	*SD*
Five Factor Mindfulness Questionnaire Total Describe Mean Aware Mean Observe Mean Nonreact Mean Nonjudge Mean	77.34 3.38 2.85 3.74 3.59 2.66	10.93 .67 1.02 .72 .76 .85
General Health Questionnaire Total	19.67	4.91
Impact of Events Scale Total	44.63	23.50
Intolerance of Uncertainty Inventory Total	39.81	9.88
Patient Health Questionnaire Total	10.44	7.60
Perceived Susceptibility to COVID-19	2.85	1.06
Perceived Vulnerability to Disease Total	46.72	6.02
Preventive Action Taken Scale PPE Total	8.09	2.45
Preventive Action Taken Scale Avoid Travel and Contact with Other People Total	15.89	3.05
Quality of Life Inventory Total	35.00	5.81

**Table 4:** Descriptive statistics of the Time 1 – Time 2 Dataset (n = 151).

	Time 1		Time 2	
Measure	*M*	*SD*	*M*	*SD*
Five Factor Mindfulness Questionnaire Total	82.26	12.77	82.13	13.09
General Health Questionnaire Negative Total	18.42	5.03	18.23	4.93
Impact of Events Scale Total	33.45	25.04	51.97	24.99
Intolerance of Uncertainty Inventory Total	36.15	11.07	46.36	7.51
Patient Health Questionnaire Total	6.98	6.50	22.26	6.87
Perceived Susceptibility to COVID-19	2.72	.93	2.70	0.92
Perceived Vulnerability to Disease Total	46.72	6.02	46.10	7.92
Preventive Action Taken Scale PPE Total	7.14	2.69	7.70	2.59
Preventive Action Taken Scale Avoid Travel and Contact with Other People	16.68	3.23	16.05	3.55
Quality of Life Inventory Total	35.91	5.97	35.22	5.48

## 2 Experimental Design, Materials and Methods

The survey was developed by an international team of researchers in the early stages of the COVID-19 pandemic. The project was approved by the Bowling Green State University Institutional Review Board (#1562479-4). Amazon Mechanical Turk Workers were enrolled through CloudResearch. A total of 635 highly rated participants initially responded to the survey from April 9, 2020 to April 18, 2020. Participant data were deleted from the data set if any of the following were detected: (a) less than 75% of items completed (*n* = 131), duplicate IP address (*n* = 26), (b) failing 2 of three attention check items (*n* = 27), or (c) an unusually long time to complete the survey (*n* = 1). This resulted in a final total sample of 450 participants for the Time 1 dataset.

A follow-up survey announcement and link were sent to all 635 participants who responded to the first survey announcement. The follow-up announcement and link were posted on MTurk on June 19, 2020 and were kept active for four weeks. A total of 178 MTurk workers completed the follow-up survey (39% response rate relative to the valid 450 responses to the first survey). Participant data were examined and retained using the same methods described above for Time 1 data. Time 1 and Time 2 responses were matched using unique ID codes that participants generated at time 1, IP addresses, and demographic characteristics. Out of the 178 completed surveys, we were able to unambiguously match 151. Thus, the final sample size for the Time 1 - Time 2 dataset set is 151.

**Fig. 1:**
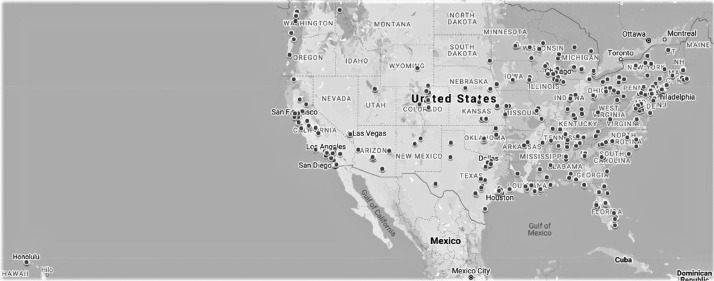
Geographic distribution of Time 1 Dataset using Google MyMaps on August 5, 2020.

**Fig. 2:**
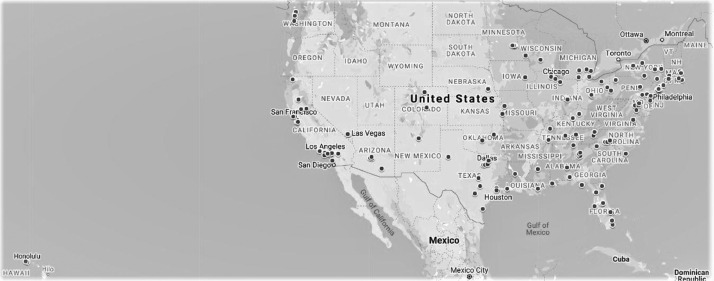
Geographic distribution of Time 1 – Time 2 Dataset using Google MyMaps on August 5, 2020.

The survey constructs and measure descriptions are provided in Table 1. The demographic characteristics of participants in the Time 1 dataset and the Time1 - Time2 dataset are summarized in Table 2. The descriptive statistics of measures in the two datasets are presented in Table 3. The geographic distribution of participants in the two datasets are presented in Figs. 1 and 2.

We comment here on the representativeness of MTurk samples. Mturk samples can have characteristics that differ from other samples used in psychological and social science research. MTurk samples tend to be more representative of the USA population relative to convenience samples, undergraduate samples, and samples collected in university communities but less representative than carefully collected national probability samples [1,2]. However, Chandler and Shapiro [2] noted that national probability samples can be biased because they rely on telephone methods that over sample older and more conservative participants.

There are strengths to MTurk samples that are important for this dataset. Most importantly, during the COVID-19 lockdown, online data gathering was the *only* practical way to gather complex survey data from diverse populations and differing geographic locations. Additionally, we adopted “best practices” by screening for response quality, using attention checks, and making sure that signaling cues were not in the surveys [3,4].

## 3 Measures

*Measure Translation.* An international team of researchers from the USA, China, and Thailand developed the survey. Measures that were originally in Mandarin were translated to English. The first author and USA researchers then independently collected data using Qualtrics and MTurk.

*Demographics.* Participants completed items that provided information about basic demographic characteristics: age, sex, race, marital status, religion, educational attainment, if they had children, living arrangements, and employment (e.g., job type, hours per week, changes in job since COVID-19). Participants also reported whether they were experiencing any illnesses or taking any medications. The demographic questionnaire used USA census items for sex, race, and religion which were then combined with items used in previous mindfulness and health investigations [5,6].

*Self-isolation.* Participants reported whether they were engaged in self-isolation or quarantine (yes/no). They also reported the number of times they left their residence in a typical day.

*COVID-19 testing and COVID-19 status.* At Time 2, participants reported whether they had a nasal and/or antibody test for COVID-19. They also reported whether or not they were infected with COVID-19 with 5 items: (a) currently not infected but not tested, (b) currently not infected based on negative test result, (c) currently infected but not tested, (d) currently infected based on positive test, (e) infected in the past based on antibody test.

*General perceived vulnerability to disease (PVD).* The PVD is a 15-item scale designed to measure general perceptions of risk for illness [7]. Items are rated on a 5-point Likert scale ranging from “strongly disagree” to “strongly agree.” A total score was calculated with higher scores indicating higher levels of perceived vulnerability.

*Perceived susceptibility to COVID-19.* A single item was constructed to assess perceived vulnerability to COVID-19. Perceived susceptibility items like this have been extensively used in the preventive health behavior literature. The item was worded “How likely is it that you will contract COVID-19?” Response options ranged from “no chance” to “certain” using a 5-point scale. The use of a single-item perceived susceptibility measure has been well supported in the preventive behavior literature [8].

*Mindfulness - Five Facet Mindfulness Questionnaire (FFMQ-24).* The FFMQ-24 is a 24-item scale designed to measure mindfulness in daily life [9]. It has five subscales: awareness, observe, describe, nonjudgment, and nonreactivity. Items are rated on a 5-point scale ranging from “never or rarely true” to “very often or always true.” A total score was calculated for the FFMQ. Mean scores were calculated for each subscale (mean scores were used because the number of items varies across subscales). Higher scores indicate higher levels of mindfulness [9].

*Intolerance of uncertainty.* The 12-item version of the intolerance of uncertainty scale [10] was used to assess psychological distress associated with ambiguity and unpredictability (e.g., “unforeseen events upset me greatly”). Items were rated using a 5-point scale ranging from “not at all characteristic of me” to “entirely characteristic of me.” A total score was calculated with higher scores indicating more intolerance of uncertainty.

*Preventive actions taken scale (PATS). T*he PATS was developed in late January 2020 based on recommendations generated by then-available COVID-19 research findings. The original 12-item measure assessed the extent to which participants engaged in behaviors to prevent COVID-19 infection. Two items were removed because of limited relevance to USA populations. Factor analyses using Time 1 data indicated that two additional items could be removed resulting in an 8-item measure. Further, a varimax factor analysis indicated that the survey measured two distinct sets of COVID-19 preventive actions: “Personal protective equipment use” (3 items) and “avoiding travel and contact with other people (5 items).” Items were rated using a 5-point scale that ranged from “does not apply at all” to “applies very much or most of the time.” Total scores for the personal protective equipment use and avoiding travel and contact with other people. Higher scores indicated more personal protective equipment use.

*Post-traumatic stress symptoms.* The Impact of Events Scale – Revised was used to measure post-traumatic stress symptoms [11]. The scale contains 22 items (e.g. “I thought about it when I didn't mean to”) that were responded to on a 5-point scale that ranged from “not at all” to “extremely.” A total score was calculated with higher scores indicating more post traumatic symptoms.

*Physical symptoms.* The Patient Health Questionnaire is a 15-item scale that [12] was used to measure physical symptoms associated with stress. The item measuring menstrual symptoms was removed because is only applied to males. Items are rated using a 3-point scale that ranges from “not bothered” to “bothered a lot.” A total score was calculated with the remaining 14 items. Higher scores indicating more symptoms.

*Psychological Symptoms of Distress.* The six negatively worded items of the General Health Questionnaire were used to measure anxiety, depression, strain, and loss of confidence [13]. The items used a 5-point scale that ranged from “much less than usual” to “much more than usual.” A total score was calculated for this measure with higher scores indicating more distress.

*Quality of Life. T*en items were taken from the 26 item World Health Organization Quality of life Brief scale [14]. Items were responded to using 5-point scales with anchors such as “never” to “very often” and “very dissatisfied” to “very satisfied.” The items measured: life, health, sleep, work, self, concentrate, everyday life, depression, enjoy life, life meaning. A total score was calculated for this measure with higher scores indicating better quality of life.

## 4 Procedure

An announcement was placed on Amazon Mechanical Turk on April 9, 2020. The announcement read “The COVID-19 situation is creating worldwide challenges. University researchers hope to gain important useful information about how people are reacting to COVID-19 and coping with COVID-19. The survey is intended to be taken by individuals who are at least 18 years old who reside in the United States. The survey should take around 20 min to complete. Follow-up surveys will be sent to participants in the future to measure possible changes across time. You will receive $1.00 for completing each survey.” Interested participants were then directed the survey informed consent form. If the participant provided consent, they were linked to the survey. The survey contained 3 attention check items and 3 captcha items. If a participant skipped an item, they were asked if they intended to skip the item(s) before being able to move on to the next page of the survey. If the participant responded “yes” that they intended to skip an item, they could move on to the next page of the survey. If they responded “no” they were returned to the skipped item.

The follow-up survey link was sent to all participants who completed the first survey. Both surveys took about 20 min to complete. The surveys were completed anonymously.

## Ethics Statement

The project conformed to APA ethical guidelines and was approved by the Bowling Green State University Institutional Review Board. Participants were provided with informed consent. Survey items were designed to provide information on demographic characteristics, health history, risk for COVID-19, psychological distress, and preventive health behaviors. None of the survey items presented participants with stress-inducing stimuli. Therefore, there was no foreseeable risk for participation. Participants could end participation at any time without penalty. All collected data were not linked to participant identifying information or identifiable information. Therefore, responses were anonymous which was designed to provide participants with assurance of a higher degree of confidentiality.

## CRediT Author Statement

**William H. O'Brien:** Conceptualization, methodology, validation, formal analysis, investigation, resources, data curation, writing-original draft, writing-review and editing, supervision, project administration, funding acquisition. **Shan Wang:** Conceptualization, methodology, validation, investigation, resources, data curation, writing-original draft, writing-review and editing, supervision, project administration. **Huanzhen Xu:** Conceptualization, methodology, software, validation, investigation, resources, writing-original draft, writing-review and editing, project administration. **Zaiying Yang:** Conceptualization, methodology, investigation, resources, writing-review and editing. **Joy Ting Yang:** Methodology, resources, writing-review and editing, supervision, project administration. **Qinwanxian Liu:** Conceptualization, methodology, validation, investigation, writing-review and editing. **Xin Zhang:** Conceptualization, methodology, software, validation, investigation, writing-original draft, writing-review and editing, project administration. **Lingli Tang:** Methodology, software, validation, writing-review and editing, visualization, project administration. **Aniko V. Varga:** Conceptualization, methodology, software, validation, investigation, writing-original draft, writing-review and editing, project administration. **Tracy Sims:** Conceptualization, methodology, software, validation, investigation, writing-review and editing, project administration. **Chung Xiann Lim:** Methodology, data curation, writing-review and editing, visualization. **Somboon Jurakasemthawee:** Conceptualization, methodology, investigation, writing-review and editing. **Kullaya Pisitsungkagarn:** Conceptualization, methodology, investigation, writing-review and editing.

## Declaration of Competing Interest

All authors declare that they have no known competing financial interests or personal relationships which have, or could be perceived to have, influenced the work reported in this article.

## Appendix Supplementary materials

Image, application 1

Image, application 2
