# Supplementary material for: Psychological reactions to COVID-19: Survey data assessing perceived susceptibility, distress, mindfulness, and preventive health behaviors
Source: Data Brief. 2020 Dec 30;34:106687. doi: 10.1016/j.dib.2020.106687 (PMC7797503; doi:10.1016/j.dib.2020.106687)
Supplement: Supplementary file 2 [file mmc2.pdf]

Patient Health Questionnaire Somatic Symptom Severity Scale (PHQ-15)

During the past month, how much have you been bothered by any of the following problems:

	Not bothered at all (1)	Bothered a little (2)	Bothered a lot (3)
Stomach pain (1)	○	○	○
Back pain (2)	○	○	○
Pain in your arms or legs or other joints (3)	○	○	○
Menstrual cramps or other problems with your periods (women only) (4)	○	○	○
Headaches (5)	○	○	○
Chest Pain (6)	○	○	○
Dizziness (7)	○	○	○
Fainting spells (8)	○	○	○
Feeling your heart pound or race (9)	○	○	○
Shortness of breath (10)	○	○	○
Pain or problems during sexual intercourse (11)	○	○	○
Constipation, loose bowels, or diarrhea (12)	○	○	○
Nausea, gas, or indigestion (13)	○	○	○
Feeling tired, or having low energy	○	○	○

(14)			
Trouble sleeping (15)	○	○	○

Five Factor Mindfulness Questionnaire (FFMQ)

Below is a collection of statements about your everyday experience. Using the 1-5 scale below, please indicate, in the box to the right of each statement, how frequently or infrequently you have had each experience in the last month. Please answer according to what really reflects your experience rather than what you think your experience should be.

	never or very rarely true (1)	not often true (2)	sometimes true, sometimes not true (3)	often true (4)	very often or always true (5)
I'm good at finding the words to describe my feelings (1)	○	○	○	○	○
I can easily put my beliefs, opinions, and expectations into words (2)	○	○	○	○	○
I watch my feelings without getting carried away by them (3)	○	○	○	○	○
I tell myself that I shouldn't be feeling the way I'm feeling (4)	○	○	○	○	○
It's hard for me to find the words to describe what I'm thinking (5)	○	○	○	○	○
I pay attention to physical experiences,	○	○	○	○	○

such as the wind in my hair or sun on my face (6)					
I make judgments about whether my thoughts are good or bad (7)	○	○	○	○	○
I find it difficult to stay focused on what's happening in the present moment (8)	○	○	○	○	○
When I have distressing thoughts or images, I don't let myself be carried away by them (9)	○	○	○	○	○
Generally, I pay attention to sounds, such as clocks ticking, birds chirping, or cars passing (10)	○	○	○	○	○
When I feel something in my body, it's hard for me to find the right words to describe it (11)	○	○	○	○	○
It seems I am "running on automatic"	○	○	○	○	○

without much awareness of what I'm doing (12)					
When I have distressing thoughts or images, I feel calm soon after (13)	○	○	○	○	○
I tell myself I shouldn't be thinking the way I'm thinking (14)	○	○	○	○	○
I notice the smells and aromas of things (15)	○	○	○	○	○
Even when I'm feeling terribly upset, I can find a way to put it into words (16)	○	○	○	○	○
I rush through activities without being really attentive to them (17)	○	○	○	○	○
Usually when I have distressing thoughts or images I can just notice them without reacting (18)	○	○	○	○	○
I think some of my emotions are bad or inappropriate and I	○	○	○	○	○

shouldn't feel them (19)					
I notice visual elements in art or nature, such as colors, shapes, textures, or patterns of light and shadow (20)	○	○	○	○	○
When I have distressing thoughts or images, I just notice them and let them go (21)	○	○	○	○	○
I do jobs or tasks automatically without being aware of what I'm doing (22)	○	○	○	○	○
I find myself doing things without paying attention (23)	○	○	○	○	○
I disapprove of myself when I have illogical ideas (24)	○	○	○	○	○

General Health Questionnaire (GHQ)

We would like to know how your health has been in general, over the past month. Please select the answer that best applies to your experience of then last month.

	Much less than usual (1)	Less than usual (2)	Same as usual (3)	More than usual (4)	Much more than usual (5)
Been able to concentrate on whatever you are doing? (1)	○	○	○	○	○
Lost much sleep over worry? (2)	○	○	○	○	○
Felt that you were playing a useful part in things? (3)	○	○	○	○	○
Felt capable of making decisions about things? (4)	○	○	○	○	○
Felt constantly under strain? (5)	○	○	○	○	○
Felt that you couldn't overcome your difficulties? (6)	○	○	○	○	○
Been able to enjoy your normal day-to-day activities? (7)	○	○	○	○	○
Been able to face up to your problems? (8)	○	○	○	○	○
Been feeling unhappy and	○	○	○	○	○

depressed? (9)					
Been losing self-confidence in yourself? (10)	○	○	○	○	○
Been thinking of yourself as a worthless person? (11)	○	○	○	○	○
Been feeling reasonably happy, all things considered? (12)	○	○	○	○	○

Perceived Vulnerability to Disease (PVD)

Please rate the extent to which you agree with each of the statements below.

	Strongly disagree (1)	Disagree (2)	Neutral (3)	Agree (4)	Strongly agree (5)
It really bothers me when people sneeze without covering their mouths. (1)	○	○	○	○	○
If an illness is 'going around', I will get it. (2)	○	○	○	○	○
I am comfortable sharing a water bottle with a friend. (3)	○	○	○	○	○
I don't like to write with a pencil someone else has obviously chewed on. (4)	○	○	○	○	○
My past experiences make me believe I am not likely to get sick even when my friends are sick. (5)	○	○	○	○	○
I have a history of susceptibility to infectious diseases. (6)	○	○	○	○	○

I prefer to wash my hands pretty soon after shaking someone's hand. (7)	○	○	○	○	○
In general, I am very susceptible to colds, flu, and other infectious diseases. (8)	○	○	○	○	○
I dislike wearing used clothes because you don't know what the past person who wore it was like. (9)	○	○	○	○	○
I am more likely than the people around me to catch an infectious disease. (10)	○	○	○	○	○
My hands do not feel dirty after touching money. (11)	○	○	○	○	○
I am unlikely to catch a cold, flu, or other illness, even if it is going around. (12)	○	○	○	○	○
It does not make me anxious to be around sick people. (13)	○	○	○	○	○

My immune system protects me from most illnesses that other people get. (14)	○	○	○	○	○
I avoid using public telephones because of the risk that I may catch something from the previous user. (15)	○	○	○	○	○

Intolerance of Uncertainty Scale (IOUS)

Please rate how much you agree with each item:

	Not at all characteristic of me (1)	A little characteristic of me (2)	Somewhat characteristic of me (3)	Very characteristic of me (4)	Entirely characteristic of me (5)
Unforeseen events upset me greatly. (1)	○	○	○	○	○
It frustrates me not having all the information I need. (2)	○	○	○	○	○
Uncertainty keeps me from living a full life. (3)	○	○	○	○	○
One should always look ahead so as to avoid surprises. (4)	○	○	○	○	○
A small unforeseen event can spoil everything, even with the best of planning. (5)	○	○	○	○	○
When it's time to act, uncertainty paralyses me. (6)	○	○	○	○	○
When I am uncertain I can't function very well. (7)	○	○	○	○	○
I always want to know what the future has	○	○	○	○	○

in store for me. (8)					
I can't stand being taken by surprise. (9)	○	○	○	○	○
The smallest doubt can stop me from acting. (10)	○	○	○	○	○
I should be able to organize everything in advance. (11)	○	○	○	○	○
I must get away from all uncertain situations. (12)	○	○	○	○	○

Preventive Action Taken Scale (PATS)

Please read each statement and rate how much the statement applies to you. There are no right or wrong answers. Do not spend too much time on any statement.

	Does not apply to me at all (1)	Applies to me to some degree, or some of the time (2)	Applies to me to a considerable degree or a good part of time (3)	Applies to me very much or most of the time (4)
I engage in precautionary purchases of masks, medicinal alcohol, gloves, medicine or treatment for flu. (1)	○	○	○	○
I avoid public events and crowded places. (2)	○	○	○	○
I avoid using public transport. (3)	○	○	○	○
I avoid going to highly affected places, such as New York or Washington at the moment. (4)	○	○	○	○
I avoid physical contact with other people. (5)	○	○	○	○
I increase my hygiene behavior (e.g. wash my hands more often) (6)	○	○	○	○
I wear a face mask outside of my home. (7)	○	○	○	○
I wear a face mask	○	○	○	○

everywhere. (8)				
I wear gloves outside of my home. (9)	○	○	○	○
Please answer "applies to me very much or most of the time" for this item (10)	○	○	○	○
I wear gloves everywhere. (11)	○	○	○	○
I avoid eating any wild animal. (12)	○	○	○	○
I avoid eating any animal meat (pork, chicken, beef, ect.). (13)	○	○	○	○

Impact of Events Scale (IOES)

The following is a list of difficulties people sometimes have after stressful life events. Please read each item, and then indicate how distressing each difficulty has been for you ***during the past 7 days*** with respect to the disaster. How much were you distressed or bothered by these difficulties?

	Not at all (1)	A little bit (2)	Moderately (3)	Quite a bit (4)	Extremely (5)
Any reminder brought back feelings about it. (1)	○	○	○	○	○
I had trouble staying asleep. (2)	○	○	○	○	○
Other things kept making me think about it. (3)	○	○	○	○	○
I felt irritable and angry. (4)	○	○	○	○	○
I avoided letting myself get upset when I thought about it or was reminded of it. (5)	○	○	○	○	○
I thought about it when I didn't mean to. (6)	○	○	○	○	○
I felt as if it hadn't happened or wasn't real (7)	○	○	○	○	○
I stayed away from reminders about it. (8)	○	○	○	○	○
Pictures about it	○	○	○	○	○

popped into my mind. (9)					
I was jumpy and easily startled. (10)	○	○	○	○	○
I tried not to think about it. (11)	○	○	○	○	○
I was aware that I still had a lot of feelings about it, but I didn't deal with them. (12)	○	○	○	○	○
My feelings about it were kind of numb. (13)	○	○	○	○	○
I found myself acting or feeling like I was back at that time. (14)	○	○	○	○	○
I had trouble falling asleep. (15)	○	○	○	○	○
I had waves of strong feelings about it. (16)	○	○	○	○	○
I tried to remove it from my memory. (17)	○	○	○	○	○
I had trouble concentrating. (18)	○	○	○	○	○
Reminders of it caused me to have physical reactions, such as	○	○	○	○	○

seating, trouble breathing, nausea, or a pounding heart. (19)					
I had dreams about it. (20)	○	○	○	○	○
I felt watchful and on guard. (21)	○	○	○	○	○
I tried not to talk about it. (22)	○	○	○	○	○

DEMOGRAPHICS

Age

Please enter your age: ________________________

Sex

What is your sex?

○ Male (2)

○ Female (1)

○ Other (3) ____

Employment COVID

Which of the following best describes your CURRENT employment arrangements?

- ○ Employed, working 1-24 hours per week (1)
- ○ Employed, working 24-39 hours per week (2)
- ○ Employed, working 40 or more hours per week (3)
- ○ Not employed, looking for work (4)
- ○ Not employed, NOT looking for work (5)
- ○ Retired (6)
- ○ Disabled, not able to work (7)

Marital Status

What is your marital status?

- ○ Single (1)
- ○ Cohabiting (not married) (2)
- ○ Long term relationship (not married or cohabiting) (3)
- ○ Married (4)
- ○ Divorced (5)
- ○ Widowed (6)
- ○ Other (please specify) (7) ____

Children

Do you have children?

- ○ yes (1)
- ○ no (2)

Income

Please indicate your pretax annual income in dollars

- ○ 0 - 10,000
- ○ 10,001 - 20,000
- ○ 20,001 - 30,000
- ○ 30,001 - 40,000
- ○ 40,001 - 50,000
- ○ 50,001 - 60,000
- ○ 60,001 - 70,000
- ○ 70,001 - 80,000
- ○ 80,001 - 90,000
- ○ 90,001 - 100,000
- ○ 100,001 - 120,000
- ○ 120,001 - 140,000
- ○ 140,001 - 160,000
- ○ 160,001 - 180,000
- ○ 180,001 - 200,000

○ 200,001 +

Illnesses

Do you have any medical conditions (not coronavirus)?

○ Yes, please list (2) ____

○ No (1)

Medication

Are you currently taking any medicine?

○ Yes, please list (2) ____

○ No (1)

Height

What is your height?

○ Feet ____

○ Inches ____

Weight

What is your weight in pounds?

____

Country

In what country are you currently staying?

____

State

In what state are you currently staying?

____

City

In what city are you currently staying?

____

Accommodation

What type of accommodation are you currently staying?

- ○ Own home/apartment (1)
- ○ Parent's home/apartment (2)
- ○ Friend's home/apartment (3)
- ○ Hotel (4)
- ○ University accommodation (5)
- ○ Other temporary home/apartments (6)
- ○ Hospital (7)
- ○ Nursing home/Assisted living (8)
- ○ Quarantine (9)
- ○ Other (please specify) (10)

Isolation

Are you currently in self-isolation?

- ○ Yes (2)
- ○ No (1)

How many times do you leave your place in a typical day?

____

Have you been anywhere in the last 14 days that you think is risky for Coronavirus?

- ○ Yes
- ○ No

If you have been to a place in the last 14 days that you think is risky for Coronavirus, please rate the level of risk.

- ○ Extremely low risk
- ○ Slight risk
- ○ Moderate risk
- ○ High risk
- ○ Extremely high risk

COVID-19 Testing

Have you been tested for COVID-19 infection using a nasal swab for active virus infection?

○ Yes

○ No

Have you been tested for COVID-19 antibodies using a blood test?

○ Yes

○ No

Perceived Susceptibility to COVID-19

How likely is it that you will contract the Coronavirus (COVID-19)? Please base your estimate on the following rating scale?

○ No chance (1)

○ Unlikely (2)

○ About equally likely to happen or not happen (3)

○ Likely (4)

○ Certain (5)

How likely is it that you already contracted the Coronavirus (COVID-19) **IN THE PAST**?

○ No chance (1)

○ Unlikely (2)

○ About equally likely to happen or not happen (3)

○ Likely (4)

○ Certain (5)

Compared to the average person in your country, how likely is it that you will contract the Coronavirus? Base your estimates on the following rating scale:

- ○ Greatly below average
- ○ Below average
- ○ About average
- ○ Above average
- ○ Greatly above average

COVID Status

Regarding Coronavirus (COVID-19), I (check all that apply):

- ☐ am not infected, but I have not been tested
- ☐ am not infected, based on negative results from a COVID-19 antibody test or nasal swab
- ☐ could be currently infected, but I have not been tested
- ☐ am currently infected based on a positive COVID-19 test result
- ☐ was not infected in the past, but I have not been tested
- ☐ could have been infected in the past, but I have not been tested
- ☐ was infected in the past based on positive results from a COVID-19 antibody test or nasal swab
